# Metallothionein-3 modulates the amyloid β endocytosis of astrocytes through its effects on actin polymerization

**DOI:** 10.1186/s13041-015-0173-3

**Published:** 2015-12-04

**Authors:** Sook-Jeong Lee, Bo-Ra Seo, Jae-Young Koh

**Affiliations:** Neural Injury Research Center, Asan Institute for Life Sciences, University of Ulsan College of Medicine, Seoul, South Korea; Department of Neurology, Asan Medical Center, University of Ulsan College of Medicine, 88, Olympic-ro 43-Gil, Songpa-Gu, Seoul 05505 South Korea; Present address: Department of New Drug Discovery and Development, Chungnam National University, 99 Daehak-ro, Yuseong, Daejeon, 34134 South Korea

**Keywords:** Amyloid beta, Endocytosis, Metallothioneins

## Abstract

**Background:**

Astrocytes may play important roles in the pathogenesis of Alzheimer’s disease (AD) by clearing extracellular amyloid beta (Aβ) through endocytosis and degradation. We recently showed that metallothionein 3 (Mt3), a zinc-binding metallothionein that is enriched in the central nervous system, contributes to actin polymerization in astrocytes. Because actin is likely involved in the endocytosis of Aβ, we investigated the possible role of Mt3 in Aβ endocytosis by cortical astrocytes in this study.

**Results:**

To assess the route of Aβ uptake, we exposed cultured astrocytes to fluorescently labeled Aβ_1–40_ or Aβ_1–42_ together with chloropromazine (CP) or methyl-beta-cyclodextrin (MβCD), inhibitors of clathrin- and caveolin-dependent endocytosis, respectively. CP treatment almost completely blocked Aβ_1–40_ and Aβ_1–42_ endocytosis, whereas exposure to MβCD had no significant effect. Actin disruption with cytochalasin D (CytD) or latrunculin B also completely blocked Aβ_1–40_ and Aβ_1–42_ endocytosis. Because the absence of *Mt3* also results in actin disruption, we examined Aβ_1–40_ and Aβ_1–42_ uptake and expression in *Mt3*^*−/−*^ astrocytes. Compared with wild-type (WT) cells, *Mt3*^*−/−*^ cells exhibited markedly reduced Aβ_1–40_ and Aβ_1–42_ endocytosis and expression of Aβ_1-42_ monomers and oligomers. A similar reduction was observed in CytD-treated WT cells. Finally, actin disruption and *Mt3* knockout each increased the overall levels of clathrin and the associated protein phosphatidylinositol-binding clathrin assembly protein (PICALM) in astrocytes.

**Conclusions:**

Our results suggest that the absence of *Mt3* reduces Aβ uptake in astrocytes through an abnormality in actin polymerization. In light of evidence that Mt3 is downregulated in AD, our findings indicate that this mechanism may contribute to the extracellular accumulation of Aβ in this disease.

## Background

Alzheimer’s disease (AD) is the most common cause of dementia throughout the world. Pathological characteristics of AD include amyloid plaques, neurofibrillary tangles and neuronal loss. In most developed countries, the number of AD patients is rapidly increasing in parallel with the increase in aging populations. As a slowly progressive disorder that affects cognition, AD imposes enormous clinical, economic and psychological burdens on families and societies, but unfortunately, no disease-modifying treatment for AD is currently available.

Although diverse pathogenic mechanisms for AD have been proposed, most research has focused on the roles of amyloid beta (Aβ) and hyperphosphorylated tau — the main components of plaques and tangles, respectively [1]. Aβ plaques are dense and mostly insoluble deposits of Aβ protein fragments that build up outside and around neurons. In contrast, neurofibrillary tangles are twisted fibers of tau proteins that accumulate inside nerve cells [1]. These abnormal protein aggregates disturb neuronal networks in the brain, eventually resulting in synaptic malfunction and neuronal loss. Of the two, Aβ may be a more proximal cause of AD because mutations in the amyloid precursor protein (APP) gene are sufficient to cause AD.

APP is a plasma membrane protein whose physiological functions are not yet clear [2–4]. Generating Aβ from APP requires sequential β- and γ-proteolytic cleavages inside cells [5, 6]. Once it has been formed inside neurons, Aβ is secreted to the extracellular space. The extracellular Aβ level is determined by the Aβ secretion rate and the rate of Aβ clearance through endocytosis and degradation [7, 8]. Accordingly, alterations in the endocytosis of neuronal APP affect the Aβ level in the brain [9, 10]. Astrocytes produce low amounts of Aβ [11]. Therefore, the intracellular pool of Aβ in astrocytes could be derived primarily from increased internalization of the exogenous Aβ generated by neurons, suggesting that astrocytes may be the main contributors to the clearance and degradation of Aβ peptide [11, 12].

The cytoskeletal protein actin regulates various cellular functions. It dynamically forms a polar filamentous structure (F-actin) that associates with diverse proteins [13, 14]. Actin bundles localized under the plasma membrane play a key role in the endocytosis of external signaling-molecule complexes [15, 16]. In particular, actin dynamics affect clathrin-mediated endocytosis [17, 18]. Actin bundles interact with invaginated clathrin-coated pits, pulling them into the intracellular space [18]. Internalized vesicles associated with diverse proteins are then sent to their final destinations, such as lysosomes or Golgi bodies, and are thereby degraded or recycled. Therefore, the actin cytoskeleton has a key role in both membrane signal transduction and the metabolism of membrane proteins and their ligands, including APP and Aβ peptides. In addition to having a role in endocytosis, actin contributes to synaptic stability [19, 20]. An increasing body of evidence suggests a role for actin modulatory proteins, such as cofilin and drebrin, in AD pathology [21, 22].

Metallothionein 3 (Mt3), a zinc-binding protein enriched in the central nervous system (CNS) [23, 24], interacts with a variety of proteins, including actin [25]. Previously, we found that Mt3 plays an important role in c-Abl activation downstream of the epidermal growth factor receptor in cultured astrocytes through its effects on actin dynamics [26]. Thus, the absence of *Mt3* results in a defect in actin polymerization [26]. This finding raises the possibility that Mt3 may contribute to Aβ endocytosis processes mediated by actin polymerization, such as clathrin-dependent endocytosis. Significant downregulation of Mt3 has been shown in AD brains; therefore, such a reduction in astrocytic Aβ uptake could contribute to the accumulation of extracellular Aβ [27]. Thus, in the present study, we assessed the role of Mt3 in the endocytosis of Aβ by astrocytes.

## Results

### Clathrin-dependent endocytosis and Aβ uptake in cultured cortical astrocytes

**Fig. 1:**
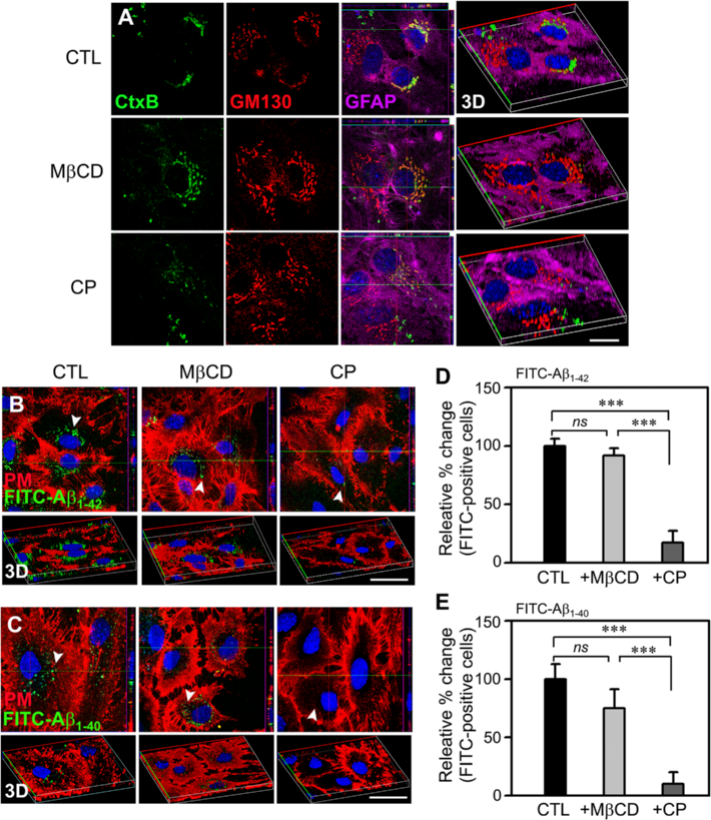
Aβ uptake in astrocytes occurs mainly through clathrin-dependent endocytosis. **a** Confocal fluorescence micrographs of WT (*Mt3* ^+/+^) astrocytes treated with 20 nM FITC-CtxB for 30 min at 37 °C. Cells were pretreated with vehicle, 1 μM CP, or 1 mM MβCD for 30 min before and during exposure to CtxB. After fixation, the cells were immunostained for the Golgi marker GM130 (*red*) and for the astrocyte marker GFAP (*purple*). Hoechst 33342 (*blue*) was used to counterstain the nuclei. CtxB uptake was disrupted by CP but not by MβCD, consistent with the involvement of the clathrin-dependent endocytotic pathway (*n* = 4 experiments). 3D images were added to help visualize the endocytosed CtxB (3D). All imaging experiments of the study were performed four times with different cultures. Scale bar, 20 μm. **b**, **c** Confocal fluorescence micrographs of cultured cortical astrocytes treated with FITC-Aβs. Cells treated with or without 1 mM MβCD or 1 μM CP were incubated with 1 μM FITC-Aβ_1–42_ *(B)* or FITC-Aβ_1–40_ *(C)* (*green dots, arrowheads*) for 1 h at 37 °C and then with Alexa Fluor 594-WGA (*red*) to stain the plasma membrane. The corresponding 3D images are also presented (*bottom panels*). Scale bar, 20 μm. **d**, **e** Bars indicate the percentage of FITC(+) cells. The values represent the mean changes in the percentage of FITC-Aβ_1–42_(+) *(D)* or FITC-Aβ_1–40_(+) *(E)* cells in the CP- or MβCD-treated groups relative to control (CTL), defined as 100 % (***P < 0.001; *ns*, not significant)

Cellular endocytosis occurs through two main mechanisms: clathrin-dependent and caveolin-dependent. To differentiate these two pathways, we monitored endocytosis in astrocytes by confocal microscopy in the presence or absence of MβCD or CP, inhibitors of caveolin- and clathrin-dependent endocytosis, respectively. To confirm the specificity of this strategy, we first evaluated the endocytosis of Alexa Fluor 488-CtxB (cholera toxin subunit B), which is known to be clathrin-dependent. To this end, we preincubated astrocytes for 30 min at 37 °C with or without 1 mM MβCD or 1 μM CP before adding Alexa Fluor 488-CtxB. Although MβCD treatment did not significantly alter the endocytosis of Alexa Fluor 488-CtxB (green), which ultimately localized to Golgi bodies (GM130 fluorescence, red), CP pre-treatment almost completely prevented Alexa Fluor 488-CtxB from reaching the Golgi, with CP-treated astrocytes showing only dispersed CtxB fluorescent signals (Fig. 1a). These results confirm the selective inhibition of the clathrin-dependent endocytosis of CtxB by CP.

Because astrocytes are considered principal players in the clearance of extracellular Aβ in AD, understanding the underlying mechanism could be important for the development of effective treatment strategies. Using the same pharmacological approach described above for Alexa Fluor 488-CtxB, we investigated which endocytosis pathway was involved in the Aβ uptake by astrocytes using fluorescein isothiocyanate (FITC)-conjugated Aβ_1–40_ and Aβ_1–42_ (FITC-Aβ _1–40_, FITC-Aβ _1–42_). Astrocytes were incubated for 15 min with 1 μM each of FITC-Aβ_1–40_ and FITC-Aβ_1–42_ after a 1-h pretreatment with vehicle alone, 1 mM MβCD, or 1 μM CP. Although a number of astrocytes exhibited FITC-positive (FITC^+^) particles in their cell bodies in the cultures treated with vehicle or MβCD (Fig. 1b, arrowheads), few astrocytes in the CP-treated cultures contained intracellular FITC^+^ particles. Quantification of FITC^+^ cells showed that compared with the vehicle or MβCD treatment, the CP treatment markedly reduced the number of FITC- Aβ_1–40_^+^ cells (Fig. 1c). These results indicate that similar to the case for CtxB endocytosis, both FITC-Aβ_1–40_ and FITC-Aβ_1–42_ also mainly enter astrocytes in a CP-sensitive, clathrin-dependent manner.

### Mt3 deletion disrupts clathrin-dependent endocytosis in astrocytes

**Fig. 2:**
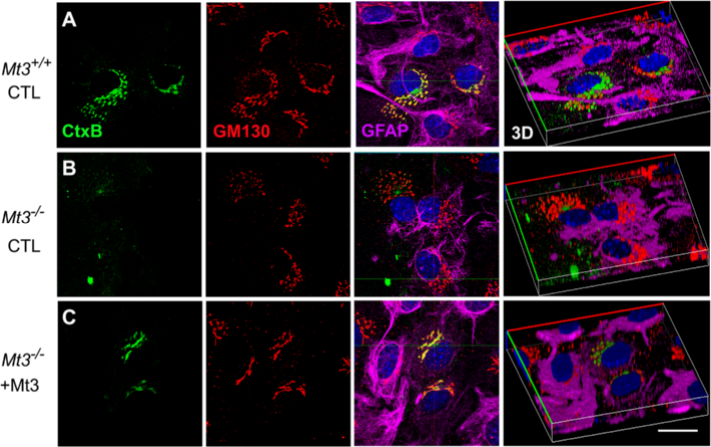
*Mt3* deletion disrupts clathrin-dependent CtxB endocytosis in astrocytes. **a**-**c** Confocal fluorescence micrographs of WT (*Mt3* ^+/+^) and *Mt3* ^*−/−*^ astrocytes treated with 20 nM FITC-CtxB for 30 min at 37 °C and of *Mt3* ^*−/−*^ astrocytes treated with 20 nM FITC-CtxB for 30 min at 37 °C in the presence of 10 μg/ml of the N-terminal Mt3 peptide *(C)*. Fixed cells were immunostained for the Golgi marker GM130 (*red*) or with GFAP (*purple*) to stain the astrocytes. In contrast to WT cells, *Mt3* ^*−/−*^ cells showed disruption of the Golgi localization of CtxB (*green*), which exhibited a dispersed distribution 30 min after uptake, but this effect was partially reversed by the Mt3 peptide treatment (10 μg/ml). Scale bar, 20 μm

We next examined the possibility that Mt3 might be involved in endocytosis. As described above, we used Alexa Fluor 488-CtxB to assess endocytosis in wild-type (WT) and *Mt3*^*−/−*^ astrocytes. Confocal microscopy showed that fluorescently labeled CtxB appeared in the Golgi body (stained with GM130) 30 min after addition in WT astrocytes (Fig. 2a). In contrast, the CtxB fluorescence in *Mt3*^*−/−*^ astrocytes appeared largely separate from the GM130 fluorescence, consistent with defective endocytosis (Fig. 2b). Furthermore, the distribution of early endosomes (EAA1 fluorescence) was more concentrated around the nuclei in *Mt3*^*−/−*^ astrocytes than in WT cells (data not shown). These findings indicate that the endocytotic process is defective in *M3t*^*−/−*^ astrocytes.

In a previous study, we reported that a fragment of Mt3, the sequence unique to Mt3 and containing a TCPCP motif at position 5–9 in the N-terminus, interacted physically with F-actin [26, 28]. Thus, we examined whether this Mt3 peptide fragment as added to the medium had any effect on CtxB uptake in astrocytes. Treatment with the N-terminal TCPCP-containing Mt3 peptide of *Mt3*^*−/−*^ astrocytes partially but not completely restored CtxB uptake (Fig. 2c).

### Mt3 deletion decreases Aβ endocytosis

**Fig. 3:**
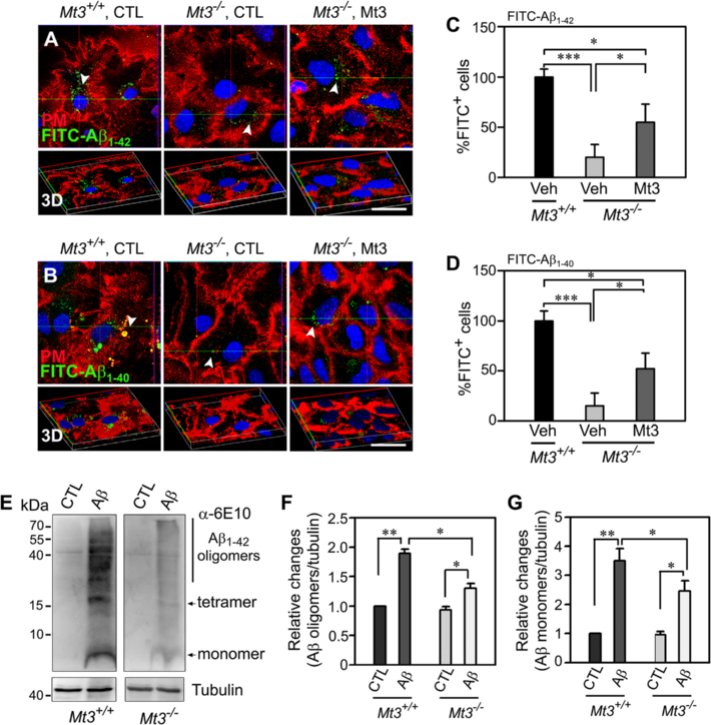
*Mt3* deletion decreases Aβ endocytosis. **a**, **b** Confocal fluorescence micrographs of WT and *Mt3* ^*−/−*^ astrocytes. Cells were incubated with 200 nM FITC-Aβ_1–42_ *(A)* or FITC-Aβ_1–40_ *(B)* (*green dots, arrowheads*) for 15 min at 37 °C. The plasma membrane was stained with Alexa Fluor 594-WGA (red). Both the FITC-Aβ_1–42_ and FITC-Aβ_1−_40 uptake were noticeably reduced in *Mt3* ^*−/−*^ cells compared with WT control cells. Again, addition of 10 μg/ml of the Mt3 peptide partly restored the endocytosis of FITC-Aβ_1–42_ and FITC-Aβ_1–40_. Scale bar, 20 μm. **c**, **d** Bars depict the percentage of FITC(+) cells in the above experiments. The values were normalized to the percentage of FITC-Aβ_1–42_^+^ *(C)* or FITC-Aβ_1–40_^+^ *(D)* cells in the WT controls, defined as 100 % (**P < 0.01 vs. WT CTL; *n* = 4 cultures). **e** Western blots for Aβ monomer and oligomers. Astrocytes from WT and *Mt3* ^*−/−*^ mice were incubated with 1 μM Aβ monomers. After 24 h, the cells were lysed and immunoblotted with an anti-6E10 antibody. **f**, **g** Bars indicate changes in the density ratio of Aβ oligomers *(F)* and Aβ monomers *(G)* relative to tubulin. All ratio values were normalized to the ratio in WT controls, which was defined as 1 (**P < 0.01 vs. WT CTL; *P < 0.05 vs. *Mt3* ^*−/−*^ CTL or WT Aβ groups; *n* = 4 experiments)

The finding that clathrin-dependent endocytosis was defective in *Mt3*^*−/−*^ astrocytes strongly suggested that Aβ endocytosis, which is clathrin-dependent, might also be defective. To examine this possibility, we first incubated *Mt3*^*−/−*^ astrocytes with 1 μM FITC-Aβ _1–40_ or FITC-Aβ _1–42_; then, the fluorescence caused by FITC-Aβ uptake was monitored for 15 min using a confocal microscope. After a 15-min incubation, WT cells already showed numerous intracellular FITC^+^ signals (Fig. 3a–d, arrowheads; *Mt3*^*+/+*^ CTL). In contrast, *Mt3*^*−/−*^ astrocytes exhibited much fewer FITC^+^ dots (Fig. 3a–d, arrowheads). As the case of CtxB uptake, when compared to *Mt3*^*−/−*^ itself, Mt3 peptide-treated *Mt3*^*−/−*^cells exhibited a slight increase in FITC^+^ signals (Fig. 3a–d). Western blot analysis using an anti-6E10 antibody also revealed that uptake of both Aβ oligomers and monomers was markedly reduced in *Mt3*^*−/−*^ astrocytes compared with WT cells (Fig. 3e–g).

### Actin disruption affects clathrin-dependent endocytosis

**Fig. 4:**
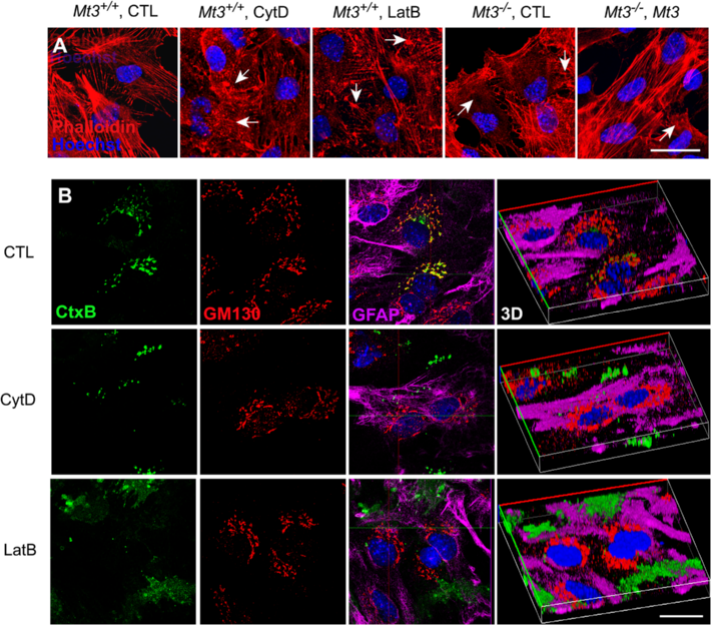
Actin disruption in *Mt3* ^*−/−*^ cells may underlie defects in clathrin-dependent endocytosis. **a** Confocal fluorescence micrographs of WT and *Mt3* ^*−/−*^ astrocytes double-stained with Alexa Fluor 633-phalloidin (F-actin; *red*) and Hoechst 33342 (nuclei; *blue*). Cells from WT and *Mt3* ^*−/−*^ mice were treated for 1 h with vehicle only (CTL) or with 100 nM CytD or 1 μM LatB or 10 μg/ml of N-terminal Mt3 peptide. After fixation, the cells were stained. Both CytD and LatB disrupted the actin cytoskeleton structure and polymerization (*arrows*). *Mt3* ^*−/−*^ astrocytes (*Mt3* ^*−/−*^, CTL) exhibited fragmented actin staining similar to that in CytD- or LatB-treated WT astrocytes, but this effect was reversed in part by treatment with the Mt3 peptide (10 μg/ml). Scale bar, 20 μm. **b** Confocal fluorescence micrographs of WT astrocytes treated with 20 nM CtxB for 30 min at 37 °C. Cells were pretreated with vehicle, 100 nM CytD or 1 μM LatB for 1 h before and during exposure to CtxB. After fixation, cells were immunostained for the Golgi marker GM130 (red) and counterstained with the nuclear dye Hoechst 33342 (*blue*). CtxB uptake was disrupted by both CytD and LatB treatment. The right column of photos represents the corresponding 3D images. Scale bar, 20 μm

Actin dynamics are important for cellular functions, such as protein endocytosis and subsequent intracellular trafficking and recycling of various cellular proteins [29, 30]. As such, alterations in actin dynamics have been linked to various neurodegenerative diseases, including AD, Parkinson’s disease and Huntington’s disease [21, 22]. Therefore, we tested whether actin disruption per se was sufficient to affect clathrin-dependent endocytosis in cortical astrocytes using the actin-disrupting drugs cytochalasin D (CytD) and latrunculin B (LatB). Moreover, to clarify whether Mt3 itself also regulated actin dynamics, 200 μg of Mt3 peptide was also added to the *Mt3*^*−/−*^ cells. The results obtained in cells pretreated with 100 nM CytD, 1 μM LatB, or Mt3 peptide were compared with those obtained in cells pretreated with vehicle. Although vehicle-treated control astrocytes exhibited a typical fibrillary pattern of actin staining (phalloidin), astrocytes treated with either inhibitor exhibited a mostly fragmented and clumped actin distribution pattern (Fig. 4a, arrows). *Mt3*^*−/−*^ cells also mimicked the effect of these inhibitors but were restored to acting in a similar manner as the vehicle-treated cells when the Mt3 peptide was added (Fig. 4a, *Mt3*^*−/−*^, *Mt3*^*−/−*^ + Mt3 peptide). In addition, to observe the actin disruption-induced CtxB uptake, pretreated astrocytes were incubated with 20 nM Alexa Fluor 488-CtxB for 30 min at 37 °C, followed by immunostaining for the Golgi marker GM130 (*red*) and imaging under a confocal microscope (Fig. 4b). Actin disruption by either drug blocked the movement of Alexa Fluor 488-CtxB into the Golgi, consistent with a defect in clathrin-dependent endocytosis. In addition, as was the case in *Mt3*^*−/−*^ astrocytes, inhibitors of actin polymerization caused early endosomes to concentrate in the vicinity of the nuclei (data not shown).

### Actin disruption per se inhibits Aβ endocytosis

**Fig. 5:**
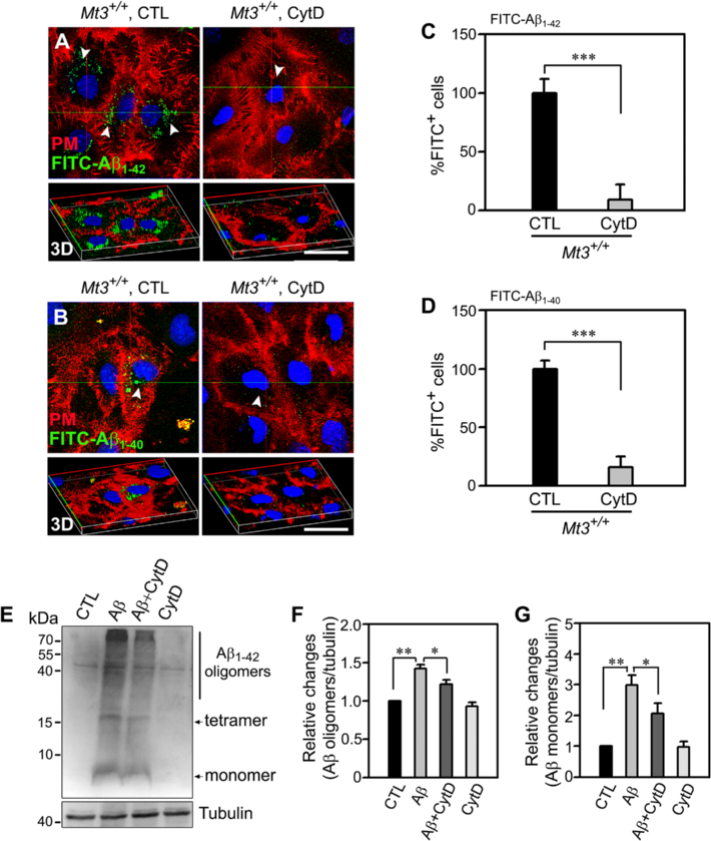
Actin disruption reduces Aβ uptake by astrocytes. **a**, **b** Confocal fluorescence micrographs of WT astrocytes were sham washed or exposed to 100 nM CytD for 1 h, then incubated with 1 μM FITC-Aβ_1–42_ *(A)* or FITC-Aβ_1–40_ *(B)* (*green dots, arrowheads*) for 15 min at 37 °C. The plasma membrane was stained with Alexa Fluor 594-WGA (*red*). Uptake of both FITC-Aβ_1–42_ and FITC-Aβ_1−_40 was noticeably reduced in CytD-treated cells compared with WT control cells. The lower panel represents the corresponding 3D images. Scale bar, 20 μm. **c**, **d** Bars depict the percentage of FITC^+^ cells. The values were normalized to the percentage of FITC-Aβ_1–42_^+^ *(C)* or FITC-Aβ_1–40_^+^ *(D)* cells in WT controls, defined as 100 % (***P < 0.001 vs. WT CTL; *n* = 4 cultures). **e** Western blots for Aβ monomers and oligomers. Astrocytes from WT mice, which were sham washed or treated with 100 nM CytD for 1 h, were incubated with 1 μM Aβ monomers. After 24 h, the cells were lysed and immunoblotted with an anti-6E10 antibody. **f**, **g** Bars indicate changes in the density ratio of Aβ oligomers (*F*) and Aβ monomers (*G*) relative to tubulin. All ratio values were normalized to the ratio in WT controls, defined as 1 (**P < 0.01 vs. WT CTL, *P < 0.05 vs. WT Aβ groups; *n* = 4 experiments)

Next, we examined whether actin disruption caused a reduction in Aβ endocytosis. As shown in Fig. 5, a number of FITC-Aβ^+^ dots were present in the cytosol of control astrocytes after a 30-min incubation; however, there were substantially fewer such dots in CytD-treated astrocytes (Fig. 5a–d). Furthermore, Western blotting analysis using an anti-6E10 antibody revealed that the uptake of both Aβ oligomers and monomers was markedly reduced in CytD-treated astrocytes compared with control cells (Fig. 5e–g).

### Mt3 deletion causes an abnormal distribution of clathrin and PICALM, an effect that is mimicked by CytD and by LatB

**Fig. 6:**
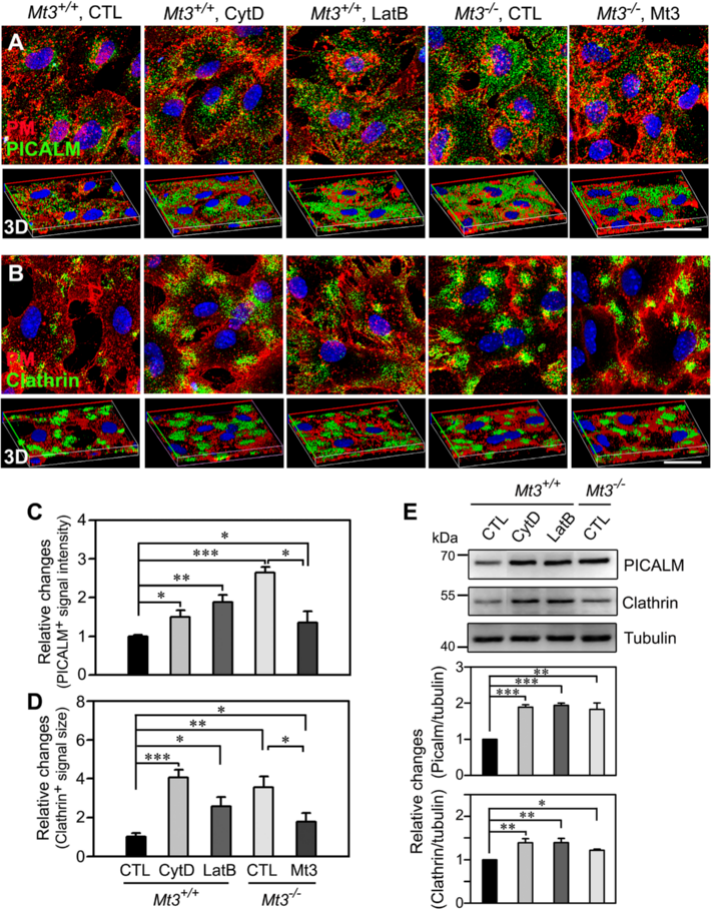
*Mt3* deletion likely causes an abnormal distribution of clathrin and PICALM through actin disruption. **a**, **b** Confocal fluorescence micrographs of WT and *Mt3* ^*−/−*^ astrocytes immunostained with an anti-PICALM antibody (*A*) or an anti-clathrin antibody (*B*). Cells from WT and *Mt3* ^*−/−*^ mice were treated for 1 h with vehicle only (CTL), 100 nM CytD, 1 μM LatB or 10 μg/ml of N-terminal Mt3 peptide, and then immunostained. The plasma membrane was stained with Alexa Fluor 594-WGA, and the nuclei were identified by counterstaining with Hoechst 33342 (*blue*). *Mt3* ^*−/−*^ cells recapitulated the CytD- and LatB-triggered alterations in the size and localization of the PICALM- and clathrin-positive signals. But, the Mt3 peptide treatment (10 μg/ml) restored just in part to the WT condition. The corresponding 3D images are shown in the lower panel. Scale bar, 20 μm. **c**, **d** Bars indicate the relative changes in the size of the PICALM *(C)*- and clathrin *(D)*-positive signals in astrocytes from WT and *Mt3* ^*−/−*^ mice treated with vehicle only (CTL), 100 nM CytD, 1 μM LatB, or 10 μg/ml of N-terminal Mt3 peptide. *Mt3* ^*−/−*^ and drug-treated WT cells showed more wide-necked, strong signals compared with those in WT controls, but Mt3 peptide-treated *Mt3* ^*−/−*^ cells still exhibited somewhat broad and intense signal (***P < 0.001, **P < 0.01, *P < 0.05 vs. WT CTL; *n* = 5 experiments). **e** Western blotting for PICALM and clathrin. WT and *Mt3* ^*−/−*^ cells were treated with vehicle, 100 nM CytD or 1 μM LatB for 1 h and immunoblotted with anti-PICALM and anti-clathrin antibodies. Bars show the density ratio of PICALM and clathrin relative to tubulin. All ratio values were normalized to the ratio in WT controls (*Mt3* ^*+/+*^ CTL), defined as 1 (***P < 0.001, **P < 0.01, *P < 0.05 vs. WT CTL; *n* = 5 experiments)

The clathrin coat machinery, of which PICALM (phosphatidylinositol-binding clathrin assembly protein) is a key component, promotes cargo recruitment and vesicle formation and is thus essential for clathrin-dependent endocytosis. Another component of this machinery is actin, which participates in the invagination of coated pits and the subsequent transport of cargo proteins to targeted cellular regions [31, 32]. Having found that the *Mt3* deletion altered actin dynamics and disturbed clathrin-mediated endocytosis, we tested whether changes in actin dynamics in *Mt3*^*−/−*^ astrocytes also modulated clathrin or PICALM expression and/or distribution. For this purpose, astrocytes from WT or *Mt3*^*−/−*^ mice were treated with 100 nM CytD, 1 μM LatB, 10 μg/ml Mt3 peptide, or vehicle for 1 h at 37 °C, after which the cells were fixed and immunostained for PICALM (green) or clathrin (green). The location of protein signals was clarified by staining the plasma membrane. Quantitative analyses of confocal images clearly demonstrated that the small-coat formation in the vicinity of the plasma membrane was strikingly altered in the drug-treated WT cells and untreated *Mt3*^*−/−*^cells, manifesting as increased intensity and stronger cytosolic or plasma membrane localization of a wide-necked-pit morphology compared with WT controls (Fig. 6a–d). In addition, the PICALM and clathrin fluorescent signals were stronger in *Mt3*^*−/−*^ cells, an effect that was mimicked by inhibition of actin polymerization in WT cells treated with CytD or LatB (Fig. 6a–d). Conversely, adding of Mt3 peptide in *Mt3*^*−/−*^ cells partly restored the *Mt3* WT pattern in terms of the localization and intensity of PICALM and clathrin (Fig. 6a–d). Consistent with this finding, Western blots showed that both PICALM and clathrin were expressed at higher levels in actin-disrupted cells than in the WT control (Fig. 6e). On the basis of these phenomena, we conclude that the absence of *Mt3* alters PICALM and clathrin activity through abnormalities in actin polymerization.

## Discussion

The central finding of the present study is that Mt3 plays a key role in the clathrin-dependent endocytosis of Aβ in astrocytes. In *Mt3*^*−/−*^ astrocytes, clathrin-mediated endocytosis, the mechanism responsible for Aβ endocytosis, was markedly decreased, whereas caveolin-mediated endocytosis was not altered. Astrocytes are likely key players in the clearance of extracellular Aβ; thus, our results suggest that changes in the Mt3 expression in astrocytes may have clinical relevance in AD. Taken together with our previous findings that Mt3 helps to maintain lysosomal degradation in astrocytes, the reduction in Mt3 in astrocytes may aggravate Aβ accumulation in the extracellular space.

Early studies showed that AD brain extracts induce more neurite outgrowth in cell cultures than do control brain extracts [27], suggesting upregulation of a growth-inducing factor or downregulation of a growth-inhibitory factor (GIF) in AD brains. The latter was shown to be the case, and a subsequent study identified the downregulated GIF as Mt3 [27, 33]. However, a later study reported controversial findings regarding the downregulation of Mt3 in AD brains [27]. Furthermore, whether Mt3 has pathogenic significance in AD has not yet been determined. Because oxidative stress may contribute to disease progression in AD [34, 35], it was postulated that reduced Mt3 expression might increase the sensitivity of neurons to oxidative injury [24, 36]. However, in our previous studies, we found that Mt3 plays a key role in autophagy/lysosomal degradation in cultured cortical astrocytes through an actin polymerization-dependent mechanism [26], suggesting a role for Mt3 in abnormal protein degradation in AD. Notably, Mt3 downregulation in AD has been reported to occur mainly in astrocytes [27, 37]. The present results suggest an additional significance for Mt3 downregulation in AD pathogenesis, namely a defect in Aβ endocytosis. Thus, Mt3-dependent defects in Aβ endocytosis and degradation may contribute to extracellular Aβ deposition in AD.

Actin polymerization plays diverse roles in cell biology. Actin fibers are involved in cell division, cell signaling, cell motility and the establishment and maintenance of the cell shape [29, 30]. In addition, actin plays an essential role in endocytosis and endosome trafficking [14, 38]. Endocytosis mainly occurs through two different mechanisms: caveolin-mediated and clathrin-mediated [39, 40]. Caveolin-mediated endocytosis is responsible for the internalization of ligands that bind to GPI-anchored proteins, lipid raft-associated receptors, cholesterol, interleukin-2 and albumin [41, 42]. In contrast, clathrin-mediated endocytosis is responsible for the internalization of low-density lipoprotein (LDL) via the LDL receptor, iron via transferrin and certain G-protein coupled receptors (GPCRs) [43–45]. A growing body of evidence indicates that the latter mechanism is dependent on actin polymerization [14, 46, 47]. CtxB endocytosis, which is clathrin-mediated, was also shown to be markedly reduced by drugs that disrupt actin polymerization in the present study. As we demonstrated previously, Mt3 interacts with actin and promotes its polymerization [26]. Consequently, in *Mt3*^*−/−*^ astrocytes, actin fibers were found to be fragmented, similar to how they were in astrocytes treated with the actin-disrupting drugs CytD and LatB. Moreover, the fact that only clathrin-mediated endocytosis is specifically reduced in *Mt3*^*−/−*^ astrocytes also strongly supports the interpretation that an actin polymerization defect underlies this phenomenon. Because Aβ endocytosis occurs largely via clathrin-mediated endocytosis, *Mt3*^*−/−*^ astrocytes were not efficient at clearing Aβ from the extracellular space.

Clathrin-mediated endocytosis requires several proteins, including PICALM, which binds to clathrin heavy chain 1 (CLTCL1). CLTCL1, in turn, is the basic subunit of the clathrin coat assembly and is thus involved in clathrin coat function during endocytosis [48, 49]. Consistent with the observed changes in clathrin-mediated endocytosis, *Mt3*^*−/−*^ and CytD- or LatB-treated astrocytes also exhibited significantly increased expression levels of both clathrin and PICALM. It is likely that the reduced endocytosis under these three conditions may have resulted in the accumulation of clathrin and PICALM in cultured astrocytes.

Our previous study demonstrated that Mt3 peptides, especially those containing the N-terminal region with the TCPCP motif, mimic Mt3 in effects on the dynamics of the actin cytoskeleton [26]. Since all changes shown in *Mt3*^*−/−*^ cells such as actin structure, CtxB uptake, localization and expression of PICALM and clathrin, and Aβ uptake were partly reversed when the TCPCP-containing Mt3 peptide was added, the Mt3 peptide might be taken up and compensate for the deficiency of endogenous Mt3 in astrocytes.

It is likely that endocytosis, endosome trafficking and lysosomal degradation are closely interrelated in cells. Actin polymerization appears to be involved in all three processes. In the case of endosome trafficking, we found that actin disruption resulted in the accumulation of early endosomes in the vicinity of nuclei. Furthermore, actin disruption completely inhibited the delivery of CtxB to the Golgi, which is an endosome-mediated process. Thus, the delivery of endosomes to their destinations (Golgi or lysosomes) may be abnormal in astrocytes in which actin polymerization is abnormal. Therefore, abnormalities in all three actin-dependent processes, as may occur in *Mt3*-null cells, may result in reduced uptake of Aβ by astrocytes.

## Conclusions

The results of the present study suggest that Mt3 plays a significant role in clathrin-dependent endocytosis, which is the main mechanism for Aβ uptake in astrocytes. Considering the potential significance of Aβ clearance in the pathogenesis of AD [50, 51], these new insights into the roles of Mt3 in Aβ endocytosis and endosome trafficking may provide helpful clues for strategies to promote Aβ clearance in AD.

## Methods

### Chemicals and antibodies

FITC-conjugated Aβ_1–40_ and Aβ_1–42_, Alexa Fluor 488-conjugated recombinant CtxB (Alexa Fluor 488-CtxB), Alexa Fluor 594-conjugated WGA (Alexa Fluor 594-WGA), Alexa Fluor 633-conjugated phalloidin (Alexa Fluor 633-phalloidin), Alexa Fluor 555-conjugated donkey anti-rabbit IgG (H + L), and Hoechst 33342 were purchased from Invitrogen (Carlsbad, CA, USA). Mt3 peptide (N-terminal region; MDPETCPCPTGGSCTCSDKC) was synthesized by Peptron (Daejeon, Korea). Antibodies against GM130 and PICALM were obtained from Abcam (Cambridge, MA, USA); antibodies against GFAP (glial fibrillary acidic protein) and clathrin were purchased from Cell Signaling Technology (Danvers, MA, USA) and Santa Cruz Biotechnology (Dallas, TX, USA), respectively. CP, an inhibitor of clathrin-dependent endocytosis, and MβCD, which removes or disrupts cholesterol in plasma membranes, were purchased from Sigma-Aldrich (St. Louis, MO, USA). CytD and LatB, which disrupt actin, were also obtained from Sigma-Aldrich.

### Animals

*Mt3*^−/−^ mice, produced as described by Erickson et al. [52], were kindly provided by Dr. Palmiter (University of Washington, Seattle, USA). *Mt3*^+/+^ wild-type (WT) mice and *Mt3*^−/−^ mice were obtained by breeding male heterozygous C67BL6/129Sv hybrid mice (*Mt3*^+/−^) with female heterozygous mice. The mice were maintained in the Animal Facility of the University of Ulsan, College of Medicine (Seoul, Republic of Korea). The offspring of the matings were genotyped by polymerase chain reaction (PCR) using the WT-specific sense primer 5′-CTC TCT ACA GAG GCC CGG CAG TCA C-3′ and the primer 5′-CAC AGT CCT TGG CAC ACT TCT CAC ATC CG-3′ (for both types).

### Cell culture

Astrocytes were prepared from neonatal (2- to 3 d old) mice. Cerebral cortices were removed, triturated by pipetting and seeded into multi-well culture plates (BD Biosciences, Franklin Lakes, NJ, USA) in Dulbecco’s modified Eagle’s medium (DMEM) supplemented with fetal bovine serum (FBS) and horse serum (7 % [v/v] of each component) and with penicillin-streptomycin (100 IU/mL and 100 μg/mL, respectively). Primary cortical cultures were incubated at 37 °C in a humidified 5 % (v/v) CO_2_ atmosphere until the cells reached confluence. The medium was changed every 3 d with rigorous shaking to remove contaminating non-adherent cells from adherent astrocytes. The resulting astrocyte-enriched cultures were approximately 95 % pure, as indicated by tests for glial fibrillary acidic protein (GFAP) immunoreactivity. All culture reagents except FBS (Hyclone, Logan, UT, USA) were purchased from Invitrogen.

### Uptake of cholera toxin subunit B

After a wash with minimal essential medium (MEM), astrocyte cultures were incubated with 20 nM CtxB for 1 h at 4 °C. After reaching a steady state of CtxB binding, the washed cells were further incubated at 37 °C in a humidified CO_2_ incubator for the indicated times. After incubation, the cells were washed with cold phosphate-buffered saline (PBS) to remove unbound CtxB and fixed by incubation with ice-cold 4 % paraformaldehyde (PFA) in PBS for 30 min at 4 °C. Internalized CtxB was observed by confocal fluorescence microscopy using an LSM-710 microscope (Carl Zeiss, Oberkochen, Germany) equipped with ZEN software and a laser-imaging system with different filters.

### Preparation of Aβ oligomers

Throughout this work, Aβ_40_ or Aβ_42_ (Genscript, Piscataway, NJ, USA) was used to generate the oligomeric form according to a previously published protocol [53]. In brief, lyophilized peptides were dissolved in 1,1,1,3,3,3-hexafluoro-2-propanol (HFIP; Sigma-Aldrich, Oakville, Ontario, Canada) to ensure that the starting material was in a homogeneous non-aggregated monomeric state; then, the aliquots containing peptide were placed in polypropylene microcentrifuge tubes. HFIP was removed by evaporation, and the resulting Aβ_40_ or Aβ_42_ peptide films were stored at −80 °C. For aggregation protocols, the peptide film was reconstituted in dimethylsulfoxide to give a 5 mM Aβ_40_ or Aβ_42_ stock solution. For oligomeric conditions, DMEM/F-12 (Dulbecco’s modified Eagle’s medium: nutrient mixture F-12) without phenol red was added to bring the peptide to a final concentration of 100 μM, and the solution was then incubated at 4 °C for 24 h.

### Uptake of FITC-Aβ fragments

Primary astrocytes plated on cover slips were stained with 5 μg/ml Alexa Fluor 594-WGA for 10 min at 37 °C. Stained astrocytes were incubated with 1 μM FITC-Aβ_1–40_ or FITC-Aβ_1–42_, then chased with a FITC-Aβ_1–40_- or FITC-Aβ_1–42_-free solution; the cellular uptake of FITC-Aβ fragments was analyzed at the indicated times during the chase period using a Carl Zeiss LSM710 laser-scanning confocal microscope. For counting Aβ-internalized astrocytes, at least five fields per slip containing more than 20 cells were randomly chosen. Only cells with clear intracellular Aβ were considered Aβ-positive (Aβ^+^) cells. To examine whether the actin cytoskeleton influenced the route of Aβ internalization or extent of Aβ uptake, we treated cells with 100 nM CytD, 1 μM LatB, 1 mM MβCD, 1 μM CP, or 10 μg/ml of Mt3 peptide during the chase periods.

### Immunocytochemistry

CtxB-internalized or normal astrocytes were fixed with 4 % (v/v) PFA for 30 min and permeabilized by incubation with PBS containing 0.2 % (v/v) Triton X-100 and 1 % (w/v) bovine serum albumin (BSA) for 10 min. After a PBS wash, the astrocytes were blocked with PBS containing 1 % BSA. The cells were incubated overnight with primary antibodies against clathrin, PICALM, GFAP and GM130 at 4 °C. The next day, the cells were incubated with Alexa Fluor 488-conjugated (clathrin, and PICALM) or Alexa Fluor 555-conjugated (GM130) secondary antibodies. For astrocyte staining, the cells were co-stained with a GFAP antibody. For counterstaining of the plasma membrane and nuclei, the cells were incubated with 5 μg/ml Alexa Fluor 594-WGA (at 37 °C) and 1 μM Hoechst 33342 (Invitrogen) (at RT) for 10 min. After incubation, the cells were washed three times with PBS and slide-mounted with mounting media (Dako, Glostrup, Denmark).

### Western blot analysis

Protein expression levels were measured in whole-cell extracts of cultured cortical astrocytes. Astrocytes obtained from both WT and *Mt3*-null mice were cultured for 14–21 days in vitro (DIV) and then sonicated in lysis buffer (20 mM Tris-Cl pH 7.4, 150 mM NaCl, 1 mM EDTA, 1 mM EGTA, 1 % [v/v] Triton X-100, 2.5 mM sodium pyrophosphate, 1 μM Na_3_VO_4_, 1 μg/mL leupeptin, and 1 mM PMSF). After centrifugation, the protein concentrations in the supernatants were determined by the bicinchoninic acid (BCA) method (Thermo Scientific, Rockford, IL, USA).

For Western blotting, equal amounts of protein were separated by sodium dodecyl sulfate-polyacrylamide gel electrophoresis (SDS-PAGE) and transferred to polyvinylidene difluoride membranes. Immunoreactive proteins were visualized using an enhanced chemiluminescence kit (Pierce, Rockford, IL, USA) and protein expression was quantitatively assessed by densitometric analysis of the intensity of protein bands. All experiments were repeated at least three times using cultures from neonatal mice born from different females.

### Statistics

All of the data are presented as the means ± SEMs. For multiple comparisons among groups, one-way analysis of variance followed by a Fisher LSD post hoc test was employed. Paired *t*-tests were used to analyze differences between two groups. *P*-values < 0.05 were considered statistically significant.

## Abbreviations

AD: alzheimer’s disease
Aβ: amyloid beta
Mt3: metallothionein 3
CP: chloropromazine
MβCD: methyl-beta-cyclodextrin
CytD: cytochalasin D
WT: wild type
PICALM: phosphatidylinositol-binding clathrin assembly protein
APP: amyloid precursor protein
F-actin: filamentous actin
CNS: central nervous system
CtxB: cholera toxin subunit B
FITC: fluorescein isothiocyanate
WGA: wheat germ agglutinin
LatB: latrunculin B
GIF: growth-inhibitory factor
LDL: low-density lipoprotein
GPCRs: G-protein coupled receptors
CLTCL1: clathrin heavy chain 1
PCR: polymerase chain reaction
FBS: fetal bovine serum
GFAP: glial fibrillary acidic protein
MEM: minimal essential medium
PBS: phosphate-buffered saline
PFA: paraformaldehyde
HFIP: 1,1,1,3,3,3-hexafluoro-2-propanol
DMEM/F-12: Dulbecco’s modified Eagle’s medium, nutrient mixture F-12
BSA: bovine serum albumin
DIV: days in vitro
BCA: bicinchoninic acid
SDS-PAGE: sodium dodecyl sulfate polyacrylamide gel electrophoresis
SEMs: standard errors of the mean
LSD: the least significant difference
